# Supplementary material for: Interface instability modes in freezing colloidal suspensions: revealed from onset of planar instability
Source: Sci Rep. 2016 Mar 21;6:23358. doi: 10.1038/srep23358 (PMC4800406; doi:10.1038/srep23358)
Supplement: Supplementary Information [file srep23358-s1.doc]

**Interface instability modes in freezing colloidal suspensions: revealed from onset of planar instability**

Lilin Wang^1^, Jiaxue You^2^, Zhijun Wang2[[1]](#footnote-2)*, Jincheng Wang^2^ and Xin Lin^2*^

1-School of Materials Science and Engineering, Xi'an University of Technology, Xi'an 710048, P. R. China

2-State Key Laboratory of Solidification Processing, Northwestern Polytechnical University, Xi’an 710072, P. R. China

**Supplemental materials**

**Figures S1-S2 and Movies S1-S3**


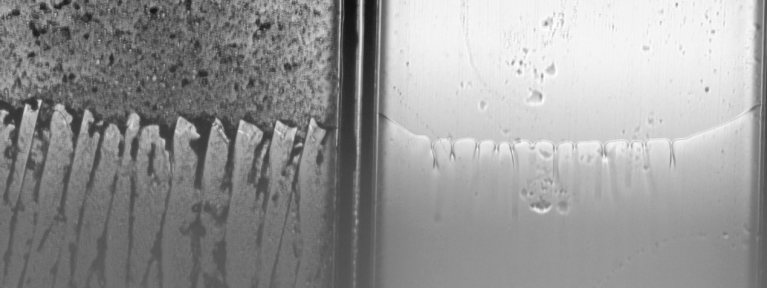

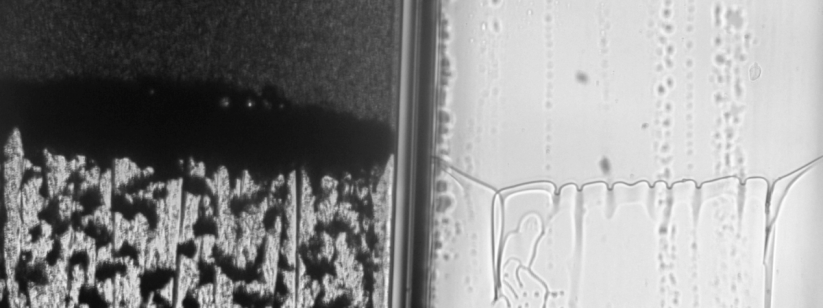

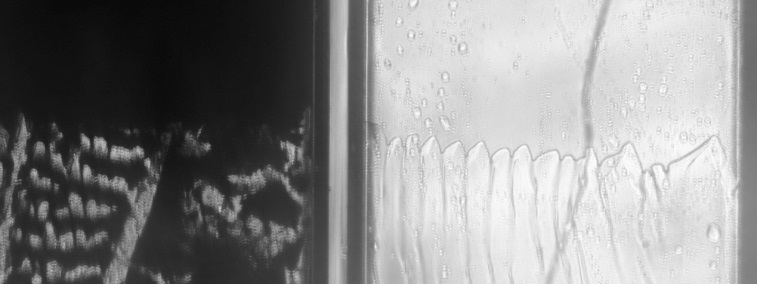


(a)

(b)

(c)

Fig. S1 steady freezing morphology of the supernatants.

For the supernatants, fluctuation of small amplitude appears on the planar interface after an incubation time, as shown in Fig. 1. The amplitude enlarged rapidly to a finite level to form cellular structure after the instability. Fig. S1(a), (b) and (c) correspond to Fig. 1(a), (b) and (c), respectively. The red scale bar is 300μm.


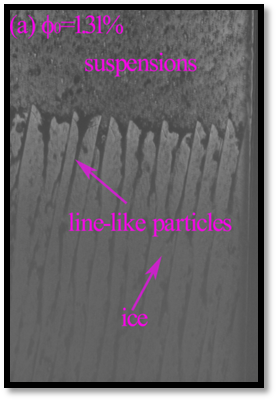

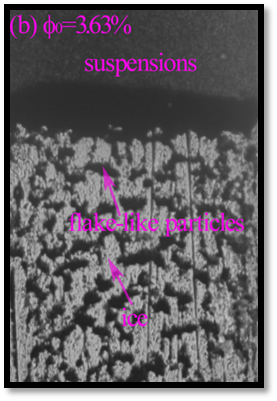

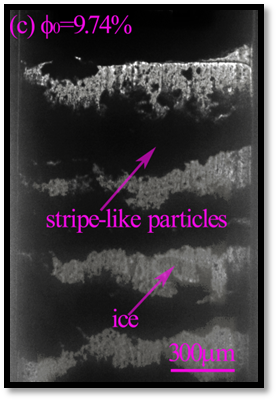


Fig. S2 steady-growth freezing morphology with different initial volume fraction of particles φ_0_ under thermal gradient G=7.23K/cm and pulling speed V=16μm/s.

The definitions of “cellular instability, local split instability and global split instability” depend on the shape of accumulated particles entrapped in the ice under steady growth. When the accumulated particles are line-like trapped between the cells (Fig.S2a), we call it as “cellular instability”. If the cumulated particles are flake-like trapped by the advancing freezing interface (Fig.S2b), it is named after “local split instability”. In a similar fashion, the stripe-like particles (Fig.S2c) is addressed as “global split instability”. The steady-growth freezing morphology of φ_0_=7.75% is very similar to that of φ_0_=9.74% (Fig.S2c). Fig.S2c (φ_0_=9.74%) is show here because it is more typical to illustrate the “global split instability”.


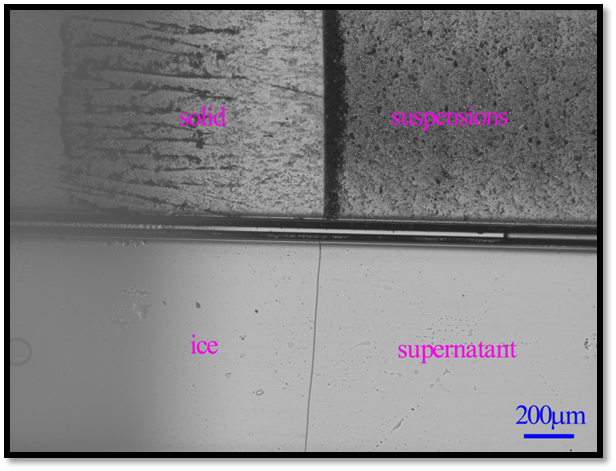


Movie S1: the dynamic initial planar instability with φ_0_=1.31%, V=16μm/s, G=7.23K/cm.


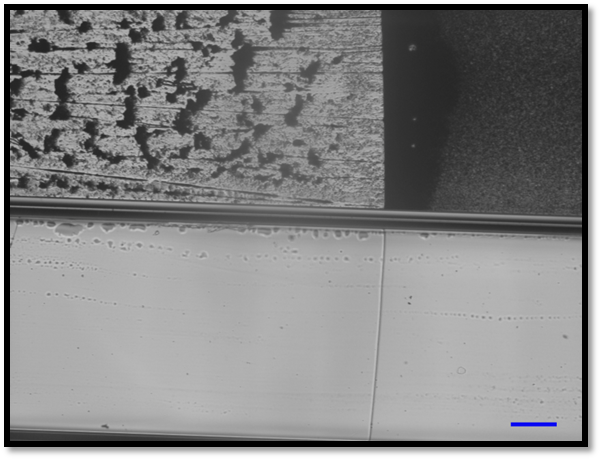


Movie S2: the dynamic initial planar instability with φ_0_=3.63%, V=16μm/s, G=7.23K/cm. The scale bar is the same as Movie S1’s.


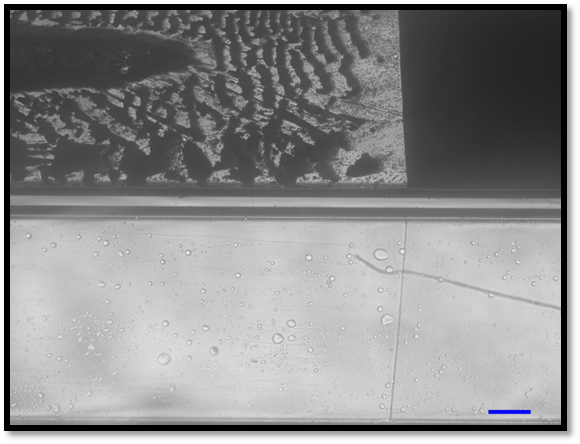


Movie S3: the dynamic initial planar instability with φ_0_=7.75%, V=16μm/s, G=7.23K/cm. The scale bar is the same as Movie S1’s.

1. ^^Corresponding author. Tel.:86-29-88460650; fax: 86-29-88491484

   E-mail address: [zhjwang@nwpu.edu.cn](mailto:zhjwang@nwpu.edu.cn) (Zhijun Wang), [xlin@nwpu.edu.cn](mailto:xlin@nwpu.edu.cn) (Xin Lin) [↑](#footnote-ref-2)
